# The Fever Epidemic

**Published:** 1920-11-06

**Authors:** 


### THE FEVER EPIDEMIC.

At present there is no sign of any marked abatement of the scarlet fever and diphtheria epidemics in this country. In the Metropolitan area at the beginning of the present week there were nearly 8,000 cases under treatment in the various fever hospitals of the Asylums Board. Speaking generally, the type of disease has been mild, and the increased prevalence this autumn is largely in accord with the seven to eight years' cycle which the virulence of these infections has shown in the past. Apart from the need for increased care of the children and the observance of customary household precautions, there is no real occasion to create alarm at the present situation, but we are glad to see, in view of the difficulty and expense of obtaining immediate skilled nursing in the average private house, that certain of the daily papers have taken pains to explain the official fever hospital procedure to the public at large.

The average citizen is curiously ignorant both of the actual organisation of the infectious fever service and of the very great advantages it confers upon both patient and relatives. Home nursing of these cases has its inevitable disadvantages to the sufferer, and the keeping of a patient at home automatically cuts off the rest of the family from association with the outside world. Notification and request from the medical attendant will, within a very short time, bring a perfectly equipped ambulance to the house. A trained nurse accompanies it, provided with all the clothing and travelling essentials for the patient, who is removed to hospital with the maximum of comfort to himself and the minimum of trouble to the household. Next day a complete disinfecting plant is brought to the spot, and, unless another case occurs in the family, most of the household disturbance is brought rapidly to an end.

This benefit to which we refer is not confined to the rate-supported institutions only. Under the Metropolitan Asylums Board there is also the London Fever Hospital, with 160 beds, at which payments by the patients are made. Certain smaller paying institutions are also available, but our point is not to recount or describe the details of these hospitals. In the hospital world they are more than well known. Simply would we point out that their invaluable organisation and assistance are ever at the public service, and that the people should realise the perfection with which these institutions carry out their work, for London has good cause to be grateful for the quiet efficiency with which it is done, and the perfection to which the treatment of the patients has been carried.

